# Six Air Pollutants Associated With Increased Risk of Thyroid Nodules: A Study of 4.9 Million Chinese Adults

**DOI:** 10.3389/fendo.2021.753607

**Published:** 2021-12-13

**Authors:** Yunjing Zhang, Kai Wang, Wei Qin, Cheng Jin, Yiqing Song, Peng Jia, Shengfeng Wang, Yongfeng Song, Yi Ning, Liming Li

**Affiliations:** ^1^ Department of Epidemiology and Biostatistics, School of Public Health, Peking University Health Science Center (PKUHSC), Beijing, China; ^2^ Department of Endocrinology, Shandong Provincial Hospital Affiliated to Shandong First Medical University, Jinan, China; ^3^ Shandong Clinical Medical Center of Endocrinology and Metabolism, Jinan, China; ^4^ Institute of Endocrinology and Metabolism, Shandong Academy of Clinical Medicine, Jinan, China; ^5^ Department of Social Medicine and Health Education, School of Public Health, PKUHSC, Beijing, China; ^6^ Meinian Institute of Health, Beijing, China; ^7^ PKUHSC Meinian Public Health Research Institute, Beijing, China; ^8^ Department of Epidemiology, Richard M. Fairbanks School of Public Health, Indiana University, Indianapolis, IN, United States; ^9^ School of Resources and Environmental Science, Wuhan University, Wuhan, China; ^10^ International Institute of Spatial Lifecourse Epidemiology (ISLE), Wuhan University, Wuhan, China

**Keywords:** thyroid nodules, PM_2.5_, PM_10_, NO_2_, SO_2_, CO, O_3_, dose–response relationship

## Abstract

**Background:**

Thyroid nodules has become a significant public health issue worldwide with a rapidly increasing prevalence. However, its association with outdoor air pollution remains poorly understood. We aim to investigate the relationship between six outdoor air pollutants (PM_2.5_, PM_10_, NO_2_, SO_2_, CO, and O_3_) and the risk of thyroid nodules.

**Methods:**

We utilized a database including 4,920,536 participants who attended the annual physical examinations in the Meinian HealthCare Screening Center in 157 Chinese cities in 2017. City-specific concentrations of six pollutants (PM_2.5_, PM_10_, NO_2_, SO_2_, CO, and O_3_) from 2015 to 2017 were estimated based on the China’s National Urban Air Quality Real Time Publishing Platform. Thyroid nodule was measured with ultrasound. Multivariable Logistic regression was used to examine the associations between air pollutants and thyroid nodules with adjustment for age, sex, education, smoking, body mass index, fasting blood glucose, triglyceride, low density lipoprotein cholesterol, high density lipoprotein cholesterol, urine iodine, gross domestic product, and thyroid stimulating hormone. We conducted stratified analyses to investigate potential effect modification by sex, age, and urine iodine groups.

**Results:**

Approximately 38% of the participants (1,869,742) were diagnosed with thyroid nodules. Each of the six air pollutants was significantly and linearly associated with the risk for thyroid nodules. The adjusted odds ratios [95% CI] for every increase of 10 μg/m^3^ for PM_2.5_, PM_10_, NO_2_, SO_2_, and O_3_ were 1.062 [1.061, 1.064], 1.04 [1.03, 1.04], 1.10 [1.09, 1.10], 1.11 [1.11, 1.12], and 1.151 [1.149, 1.154], respectively; The odds ratio for each increase of 1 mg/m^3^ for CO was 1.50 [1.49 to 1.52]. Furthermore, these associations were significantly higher in the participants who were men, younger, or having lower urine iodine level (*p <*0.001).

**Conclusion:**

The six air pollutants may contribute to the high prevalence of thyroid nodules in China.

## Introduction

Thyroid nodules (TNs) are highly prevalent worldwide with 4 to 7% by physical examinations and 30 to 67% by imaging studies (1). Although most nodules are benign, up to 20% have been found to be malignant on excision (2). Increased diagnosis of TNs may be partly due to the advancements in diagnostic technologies, especially high resolution ultrasonography, but the worldwide increase of TNs may be due to multiple factors (2), including a list of individual-level risk factors (3–5), such as sex, age, iodine intake, obesity, diabetes, dyslipidemia, and thyroid autoimmunity. Air pollution may play a significant role in the development of TNs.

Air pollutants, especially particulate matters in outdoor air pollution, are well-known human carcinogens and are increasingly associated with adverse effects on the thyroid, including disturbance of thyroid function (6–9). For example, one study with 433 pregnant women reported an inverse association between maternal exposure to PM_2.5_ and the maternal free thyroxine (FT4) levels (6); airborne particulate matter (APM), has significantly affected thyroxine binding to transthyretin (TTR) and the reduction of thyroxine (T4) level (10). Understanding the association of air pollutants and the risk of TNs has particularly important public health implications for disease prevention. Existing studies have been focused on evaluating the associations between air pollutants and thyroid dysfunction, however, in a relatively limited sample (6–9). Moreover, most participants with TNs presented morphological changes but were rarely hypothyroid/thyrotoxic (11). To the best of our knowledge, no human studies have examined the associations between major air pollutants and TNs.

Therefore, this study aimed to investigate the associations between exposure to six major air pollutants (PM_2.5_, PM_10_, NO_2_, SO_2_, CO, and O_3_) and the prevalent risk of thyroid nodules in a large health check-up population. This study has particularly important public health implications for TN prevention and air pollution control policies in all countries with serious air pollution issues and challenges.

## Methods

### Database and Study Population

**Figure 1:**
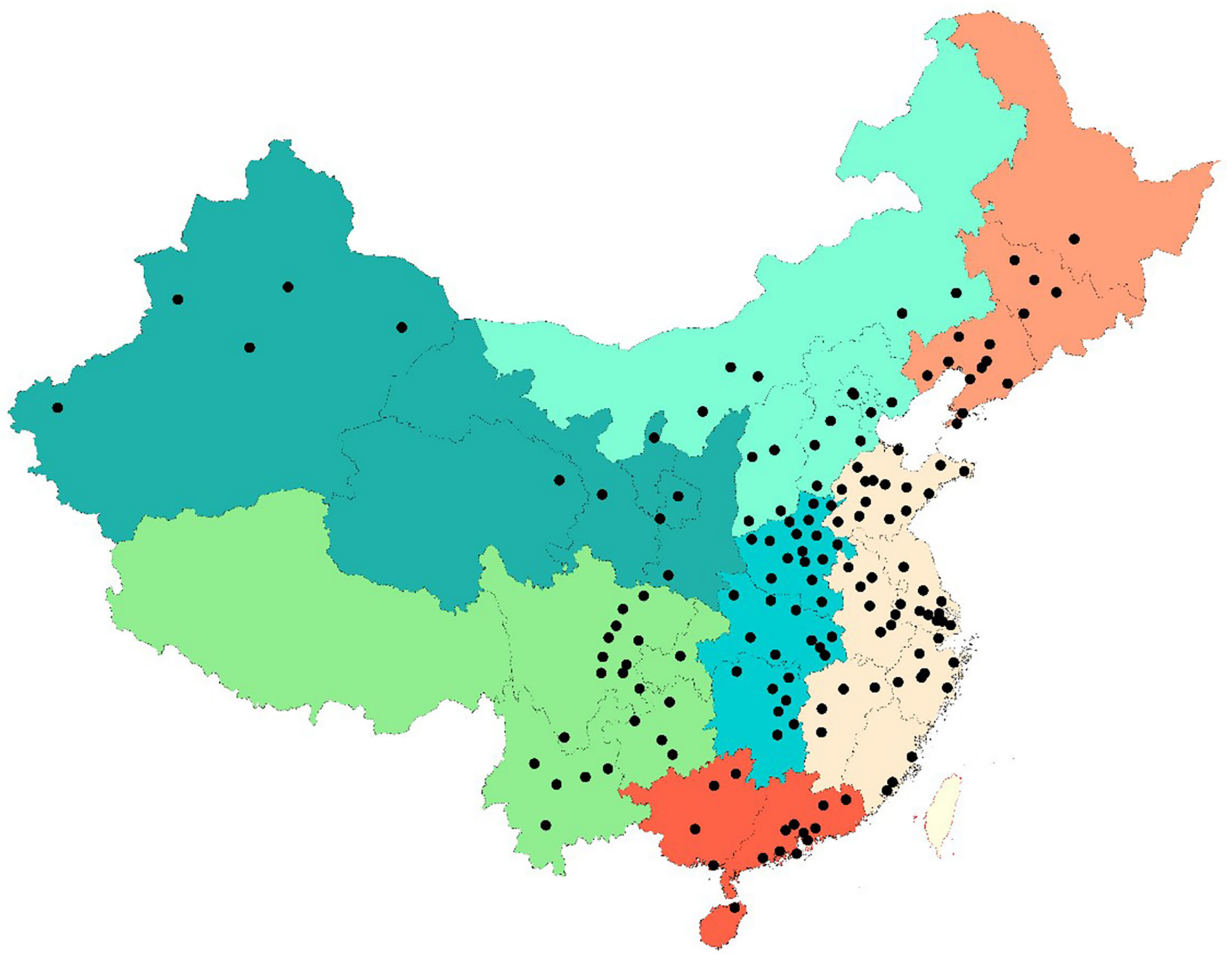
Map of participating HealthCare Screening Centers included into the analysis.

The Meinian HealthCare Screening Center is a private membership chain clinic with 319 health screening centers (**Figure 1**) covering nearly all geographic regions in mainland China (157 cities), which provides periodic health examinations to its members, mostly one yearly examination for each person. For those who attended two screenings or more, results from the most recent checkup were included, to ensure the independency of data. Every participant had signed a consent form that authorizes the Meinian Health Screening Centers to analyze the data generated from the medical screenings for academic and policy purposes. The study used the database generated from the medical screenings instead of collecting new data for the sake of the study.

The analysis was restricted to adults aged 18 years and over. A total of 4,920,536 individuals in 2017 were kept in the study without missing the value of key variables, namely, age, sex, fasting plasma glucose (FPG), body mass index (BMI), triglyceride (TG), low density lipoprotein cholesterol (LDL-C), and high density lipoprotein cholesterol (HDL-C). Data related to individual identification were removed and everyone remained anonymous during the entire study process. The project was approved by the Ethical Committee of the Health Science Center of Peking University (IRB00001052-19077).

### Measures of TNs

Ultrasound examination of the thyroid nodules was performed routinely and evaluated independently by the two senior experts with Affiniti 50 ultrasound system (Philips, Germany), which was approved by the National Center for Medical Device Evaluation (CMDE). Scans of both thyroid lobes and isthmus were obtained in both transverse and longitudinal planes. The following features were measured for each nodule: size, margin, shape, aspect ratio, capsule, hypoechoic halo, internal composition, echogenicity, presence of calcifications, vascularity on color Doppler, and cervical lymph node status.

### Measures of Air Pollutant Concentration

The average city-level annual concentrations of six air pollutants (PM_2.5_, PM_10_, NO_2_, SO_2_, CO, and O_3_) for 2015–2017 in this study were calculated based on hourly data from the China’s National Urban Air Quality Real Time Publishing Platform. The data collection and processing followed ambient air quality standards (GB 3095-2012) and the Chinese Technical Regulation for Ambient Qir Quality Assessment (HJ 663-2013). The detail methodology has been described previously (12, 13).

### Covariates

Participants were asked to provide their personal information for health history, demographic information, and medical information during the examination. BMI was calculated as weight in kilograms divided by the square of height in meters. Blood samples were drawn by venipuncture after 8–12 h of overnight fasting to measure FPG, HDL-C, LDL-C, TG, and thyroid function indexes (thyroglobulin antibodies [anti-TG], thyroid peroxidase antibody [anti-TPO], total triiodothyronine [TT3], total thyroxine [TT4], and thyroid stimulating hormone [TSH]). Serum cholesterol and triglycerides were assessed enzymatically with commercially available reagents at the clinical biochemical laboratories in each center (Roche E601, Germany). Thyroid TSH, TT3, TT4, anti-TG, and anti-TPO were measured using electrochemical luminescence method (Roche E601, Germany). FPG was grouped into three subgroups: ≤6.1 mmol/l, 6.1–6.9 mmol/l, and ≥7.0 mmol/l. BMI was divided into three levels: <24 kg/m^2^, 24–27.9 kg/m^2^, and ≥28 kg/m^2^ according to the Chinese guidelines for prevention and control of overweight and obesity (14). Serum TG, HDL-C, and LDL-C levels were classified on the basis of the Third Report of the Expert Panel on Detection, Evaluation, and Treatment of High Blood Cholesterol in Adults. The cut points for anti-TG, anti-TPO were 115 IU/ml, and 34 IU/ml, respectively.

Data about the gross domestic product per capita (GDP) and average years of education were collected from the China Statistical Yearbook for 2017. Average smoking rates were extracted from Wang et al. (15), which were estimated based on national cross-sectional surveys in 2013. The average urine iodine for each province was obtained from personal communication (TIDE program conducted by the Chinese Society of Endocrinology), which was a large-scale population-based nationwide epidemiological survey of iodine nutrition status and thyroid disease covering 31 provinces and 78,470 individuals (16).

### Statistical Analyses

The primary outcome of interest was the prevalence of thyroid nodules. Continuous variables were described as the mean ± standard deviation or median (25th–75th percentiles). The differences between participants with or without thyroid nodules were examined by *t*-tests for normally distributed data or by the Wilcoxon signed rank tests for non-normally distributed data. Categorical variables were presented as numbers and percentages, and intergroup comparisons were analyzed using Chi-square tests.

The analysis specified four logistic regression models as *a priori* to evaluate the association of thyroid nodules with air pollutants, with adjustments for an increasing number of potential confounders. The confounders were selected through careful consideration of the underlying web of causation based on the priori knowledge (3–5, 17), the availability of data in the dataset, and the univariate analysis. Model 1 included only age and sex; Model 2 added individual level variables: FPG, BMI, TG, LDL-C, and HDL-C; Model 3 added city-level variables, namely, GDP, and O_3_ concentration; and Model 4 added province-level variables: average urine iodine level, average years of education, and average smoking rate. Each air pollutant was analyzed by continuous variables, and also categorical variables divided according to the WHO air quality guideline (AQG) and data distribution of the study (18).

Stratified analyses were conducted to investigate potential effect modification by pre-specified variables: sex, age (<40, 40–59, 60–79, or ≥80 years), and urine iodine levels groups (100–199 ug/L and 200 ug/L) (19). The Bonferroni correction (nominal *P*-value divided by the number of tests), about 0.0028 for 18 tests (six pollutants ∗ three group variables), would minimize false-positive error. In sensitivity analyses, city level, TSH, and temperature were added into Model 4 to adjust their potential effect, respectively. Extended confounder models were used in sensitivity analyses because some potential effects of air pollution might be mediated by these factors (TT3 and TT4). Meanwhile, TPOAb and TGAb are two important thyroid autoantibodies, which are commonly found in patients with thyroid diseases, and have been confirmed to be two important thyroid autoantibodies to assess the risk of thyroid nodules. In order to further exclude the effect of anti-TG and anti-TPO, subgroup analysis was conducted to restrict the comparison between participants with positive in both indexes and participant with negative in both indexes.

A natural cubic spline model was utilized to study the association between each pollutant and thyroid nodules. In addition, threshold analyses were done by consecutively including participants who had exposure estimates below pre-specified thresholds in the analyses, to detect the potential minimum effective concentration for each pollutant using the method described in the study of Beelen et al. (20).

All tests were two-sided and P-values less than 0.05 were judged to be significant (except the stratified analyses). All data analyses were done in SAS 9.4 (SAS Institute Inc., Cary, NC, USA).

## Results

### Characteristics of Participants

The total study population consisted of 4,920,536 participants, of whom 1,869,742 (38.0%) were diagnosed with thyroid nodules. The individuals with thyroid nodules were more likely to be female and elderly, and had worse general health status (**Table 1**). Participants with thyroid nodules presented a lower positive rate for anti-TG and anti-TPO, had a lower level of urine iodine, but had relatively higher pollutant concentrations. Concentrations of air pollutants were varied among different cities, and the minimum number of PM_2.5_ and PM_10_ already surpassed the WHO AQG. The correlations of five air pollutants were statistically significant (*P <*0.01), except for O_3_ (*P >*0.05). PM_2.5_ was highly correlated with PM_10_ (r = 0.90) and strongly related with NO_2_ (r = 0.64) and CO (r = 0.61). CO had strong correlation with PM_10_ (r = 0.61) and SO_2_ (r = 0.63) (**Supplementary Table 1**).

**Table 1:** Comparison of basic characteristics between individuals with different thyroid nodules status (N = 4,920,536).

Characteristics	With thyroid nodules^a^	Without thyroid nodules^a^	*P*-value
N	1,869,742 (38.00)	3,050,794 (62.00)	–
Male	803,102 (42.95)	1,795,807 (58.86)	<0.0001
Age (yrs)	48 (37, 57)	37 (30, 48)	<0.0001
City scale			<0.0001
Big city	869,747 (46.52)	1,502,032 (49.23)	
Small-medium city	981,922 (52.52)	1,509,690 (49.49)	
County	18,073 (0.97)	39,072 (1.28)	
History of hypertension^b^	196,437 (12.44)	156,471 (6.07)	<0.0001
Treatment of hypertension^b^	159,529 (10.10)	112,694 (4.37)	<0.0001
Systolic pressure (mmHg)^c^	124 (112, 139)	120 (110, 131)	<0.0001
Diastolic pressure (mmHg)^c^	76 (68, 84)	74 (66, 82)	<0.0001
History of diabetes^b^	68,497 (4.34)	50,958 (1.98)	<0.0001
Treatment of diabetes^b^	51,693 (3.27)	35,571 (1.38)	<0.0001
Fasting plasma glucose (mmol/L)			<0.0001
≤6.1	1,639,756 (87.70)	2,825,449 (92.61)	
6.1–7.0	111,583 (5.97)	115,824 (3.80)	
≥7.0	118,403 (6.33)	109,521 (3.59)	
Body mass index (kg/m^2^)			<0.0001
<24	860,069 (46.00)	1,607,737 (52.70)	
24.0–27.9	720,084 (38.51)	1,043,207 (34.19)	
≥28.0	289,589 (15.49)	399,850 (13.11)	
History of dyslipidemia^b^	25,201 (1.60)	30,095 (1.17)	<0.0001
Triglyceride (mmol/L)			<0.0001
<1.7	1,305,479 (69.82)	2,173,381 (71.24)	
≥1.7	564,263 (30.18)	877,413 (28.76)	
High-density lipoprotein (mmol/L)			<0.0001
<1.03	238,922 (12.78)	428,941 (14.06)	
1.03–1.56	1,138,778 (60.91)	1,886,320 (61.83)	
≥1.56	492,042 (26.32)	735,533 (24.11)	
Low Density lipoprotein (mmol/L)			<0.0001
<2.59	824,510 (44.10)	1,486,716 (48.73)	
2.59–3.35	637,041 (34.07)	1,007,338 (33.02)	
3.36–4.13	299,364 (16.01)	416,176 (13.64)	
4.14–4.90	84,893 (4.54)	109,003 (3.57)	
≥4.91	23,934 (1.28)	31,561 (1.03)	
Total cholesterol (mmol/L)^c^	4.86 (4.25, 5.54)	4.70 (4.12, 5.36)	<0.0001
Thyroglobulin antibodies (+)^d^	8,867 (14.35)	8,436 (18.16)	<0.0001
Anti-thyroid peroxidase antibody (+)^d^	6,497 (17.78)	5,834 (20.97)	<0.0001
Total triiodothyronine (umol/l)^d^	1.5 (1.3, 1.8)	1.5 (1.3, 1.8)	<0.0001
Total tetraiodothyronine (umol/l)^d^	92.9 (75.7, 109.3)	91.2 (74.7, 107.3)	<0.0001
Thyroid Stimulating Hormone (mIU/L)^d^	1.8 (1.1, 2.9)	1.8 (1.1, 2.8)	<0.0001
PM_2.5_ (ug/m^3^)	52.4 (40.7, 66.6)	50.1 (39.5, 62.2)	<0.0001
PM_10_ (ug/m^3^)	88.4 (69.2, 113.8)	86.2 (65.0, 99.7)	<0.0001
NO_2_ (ug/m^3^)	41.7 (31.6, 45.7)	41.7 (31.4, 46.4)	<0.0001
SO_2_ (ug/m^3^)	19.0 (13.7, 29.3)	15.9 (13.1, 24.7)	<0.0001
CO (mg/m^3^)	0.98 (0.85, 1.27)	0.97 (0.83, 1.24)	<0.0001
O_3_ (ug/m^3^)	58.8 (53.7, 66.5)	57.9 (53.5, 63.1)	<0.0001
Gross Domestic Product (GDP, thousand Yuan)	81.24 (58.59, 115.05)	80.14 (61.21, 116.56)	<0.0001
Urine iodine (ug/L)	64.2 (49.8, 100.9)	72.1 (49.8 100.9)	<0.0001

a Measures of medians and quartile for continuous variables were calculated and expressed as Median (Q25, Q75). Categorical variables were summarized using the frequencies of the levels of the variables and corresponding proportions, and presented as n (%).

b There were 761,801 (15.5%) missing values for medical history, including history of hypertension, diabetes and dyslipidemia, as well as treatment of hypertension and diabetes.

c There were 7,304 (0.1%), 7,445 (0.2%), 232 (0.005%) missing values for systolic pressure, diastolic pressure, and total cholesterol, respectively.

d There were 4,812,304 (97.8%), 4,856,181 (98.7%), 3,949,760 (80.3%), 3,998,135 (81.3%), 3,688,224 (75.0%) missing values for thyroglobulin antibodies, anti-thyroid peroxidase antibody, total triiodothyronine (T3), total tetraiodothyronine (T4), and thyroid stimulating hormone (TSH), respectively.

### Association Between Air Pollutants and Thyroid Nodules

**Figure 2:**
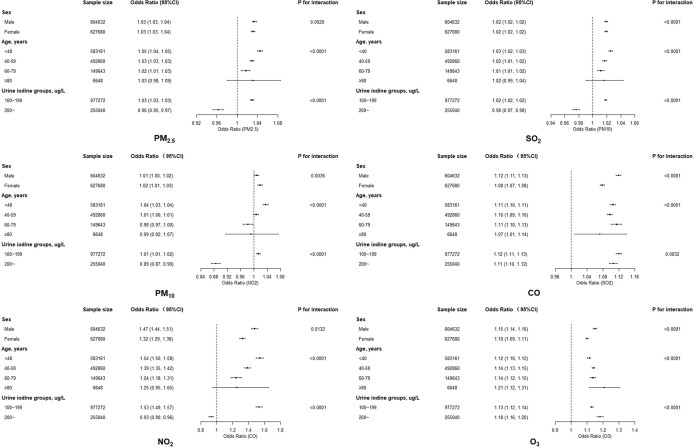
The association between exposure to six air pollutants and thyroid nodules by sex, age, and urine iodine levels.

An elevated risk was detected for exposures to any of six air pollutants in all four models (**Table 2**), with significantly adjusted ORs for each increase of 10 μg/m^3^ PM_2.5_ (1.062, 95% confidence interval [CI]: 1.061–1.064), PM_10_ (1.04, 95% CI: 1.03–1.04), NO_2_ (1.10, 95% CI: 1.09–1.10), SO_2_ (1.11, 95% CI: 1.11–1.12), O_3_ (1.151, 95% CI: 1.149–1.154), and for each increase of 1 mg/m^3^ CO (1.50, 95% CI: 1.49–1.52) in full adjusted model. All of the six pollutants also presented the statistically significant linear trends (*P-*value <0.0001). Estimates for the five types of pollutants in two-pollutant models adjusted for O_3_ (because of its weak correlation with other pollutants) did not alter the association significantly, compared to the single-pollutant models (**Table 2**). Stratified analysis indicated that ORs were relatively lower in participants who were women and elderly, and also in those having higher urine iodine level (**Figure 2** **)**. The significant positive associations for PM_2.5_, PM_10_, NO_2_, and CO were observed for participants with low urine iodine levels, but all ORs turned to be not significant within those with high iodine levels.

**Table 2:** Results of logistic regression for the association between exposure to air pollution and thyroid nodules^a^.

Model		Sample size	Model 1	Model 2	Model 3	Model 4^b^
PM_2.5_, μg/m^3^						
	Per 10 units	4,920,536	1.04 (1.04, 1.04)	1.04 (1.04, 1.04)	1.06 (1.06, 1.06)	1.06 (1.06, 1.06)
	10–29	573,013	1.00 (ref)	1.00 (ref)	1.00 (ref)	1.00 (ref)
	30–49	1,793,520	1.27 (1.26, 1.28)	1.25 (1.25, 1.26)	1.14 (1.13, 1.15)	1.16 (1.15, 1.17)
	50–69	1,824,227	1.15 (1.14, 1.16)	1.13 (1.13, 1.14)	1.21 (1.21, 1.22)	1.24 (1.23, 1.25)
	70–	729,776	1.43 (1.42, 1.45)	1.39 (1.38, 1.40)	1.46 (1.45, 1.47)	1.52 (1.51, 1.54)
PM_10_, μg/m^3^						
	Per 10 units	4,920,536	1.02 (1.02, 1.02)	1.02 (1.02, 1.02)	1.03 (1.03, 1.03)	1.04 (1.03, 1.04)
	20–39	75,311	1.00	1.00	1.00	1.00
	40–59	795,836	1.20 (1.18, 1.22)	1.20 (1.18, 1.22)	1.11 (1.09, 1.13)	1.08 (1.06, 1.10)
	60–79	1,382,917	1.47 (1.44, 1.49)	1.44 (1.42, 1.47)	1.12 (1.10, 1.14)	1.13 (1.11, 1.15)
	80–99	1,362,765	1.36 (1.34, 1.38)	1.34 (1.32, 1.37)	1.20 (1.18, 1.23)	1.22 (1.20, 1.24)
	100–	1,303,707	1.53 (1.51, 1.56)	1.49 (1.46, 1.52)	1.37 (1.35, 1.39)	1.38 (1.36, 1.41)
NO_2_, μg/m^3^						
	Per 10 units	4,920,536	1.05 (1.05, 1.05)	1.05 (1.05, 1.05)	1.09 (1.09, 1.09)	1.10 (1.09, 1.10)
	<20	98,703	1.00 (ref)	1.00 (ref)	1.00 (ref)	1.00 (ref)
	20–29	804,180	1.43 (1.41, 1.46)	1.42 (1.40, 1.44)	1.21 (1.20, 1.23)	1.24 (1.22, 1.26)
	30–39	1,281,162	1.33 (1.31, 1.35)	1.32 (1.30, 1.34)	1.17 (1.15, 1.18)	1.15 (1.14, 1.17)
	40–	2,736,491	1.46 (1.44, 1.48)	1.45 (1.43, 1.47)	1.39 (1.37, 1.41)	1.41 (1.39, 1.43)
SO_2_, μg/m^3^						
	Per 10 units	4,920,536	1.10 (1.09, 1.10)	1.09 (1.09, 1.09)	1.12 (1.10, 1.12)	1.11 (1.11, 1.12)
	<10	579,116	1.00 (ref)	1.00 (ref)	1.00 (ref)	1.00 (ref)
	10–19	2,192,717	0.98 (0.97, 0.98)	0.98 (0.98, 0.99)	1.06 (1.05, 1.06)	1.14 (1.13, 1.14)
	20–29	1,188,411	1.21 (1.21, 1.22)	1.19 (1.18, 1.20)	1.21 (1.21, 1.22)	1.31 (1.29, 1.32)
	30–	960,292	1.36 (1.35, 1.37)	1.33 (1.32, 1.34)	1.47 (1.46, 1.48)	1.55 (1.54, 1.57)
CO, mg/m^3^						
	Per 1 units	4,920,536	1.20 (1.19, 1.21)	1.17 (1.16, 1.18)	1.46 (1.45, 1.47)	1.50 (1.49, 1.52)
	0.40–0.79	676,838	1.00 (ref)	1.00 (ref)	1.00 (ref)	1.00 (ref)
	0.80–1.19	2,898,208	1.001 (0.998, 1.002)	1.001 (1.001, 1.002)	1.17 (1.16, 1.17)	1.19 (1.19, 1.20)
	1.20–	1,345,490	1.14 (1.13, 1.15)	1.12 (1.11, 1.13)	1.41 (1.40, 1.42)	1.43 (1.42, 1.44)
O_3_, μg/m^3^						
	Per 10 units	4,920,536	1.21 (1.20, 1.21)	1.20 (1.20, 1.20)	1.20 (1.18, 1.20)	1.151 (1.149, 1.154)
	<50	738,472	1.00 (ref)	1.00 (ref)	1.00 (ref)	1.00 (ref)
	50–59	2,327,055	1.17 (1.16, 1.17)	1.16 (1.16, 1.17)	1.15 (1.15, 1.16)	1.17 (1.16, 1.18)
	60–69	952,216	1.43 (1.42, 1.44)	1.40 (1.40, 1.41)	1.42 (1.41, 1.42)	1.43 (1.42, 1.44)
	70–	902,793	1.60 (1.59, 1.62)	1.58 (1.57, 1.59)	1.56 (1.55, 1.57)	1.43 (1.42, 1.44)

Data are OR (95% CI), unless indicated otherwise. ORs are presented for the following increments: 10 μg/m^3^ for PM_2.5_, PM_10_, NO_2_, SO_2_, and O_3_, and 1mg/m^3^ for CO. OR, Odds ratio.

a Model 1 included only age, and sex. Model 2 added individual level variables: fasting blood glucose, body mass index, triglyceride, low density lipoprotein, and high density lipoprotein. Model 3 added city-level variables including Gross Domestic Product, and O_3_ concentration (except for O_3_ itself). Model 4 added province-level variables including average urine iodine level, average years of education and average smoking rate.

b P-values for trend in Model 1 to Model 4 were all less than 0.05.

### Sensitivity Analyses

Only 25.0% of participants have tested TSH, and the additional adjustment for either TSH or TSH together with atmospheric temperature did not significantly change ORs compared to those for the main model, with only a small further decrease in the magnitude of ORs (**Table 3**).

**Table 3:** Sensitivity analyses results^a^.

	Odds ratio						
	Sample size	PM2.5 (ug/m^3^, Per 10 units)	PM10 (ug/m^3^, Per 10 units)	NO2 (ug/m^3^, Per 10 units)	SO2 (ug/m^3^, Per 10 units)	CO (mg/m^3^, Per 1 units)	O3 (ug/m^3^, Per 10 units)
A: Adjust the potential effect of city heterogeneity	4,920,536	1.04 (1.01, 1.08)	1.02 (1.01, 1.04)	1.12 (1.05, 1.18)	1.09 (1.05, 1.14)	1.42 (1.20, 1.68)	1.17 (1.10, 1.26)
B1: Adjusting for thyroid stimulating hormone	1,232,312	1.03 (1.03, 1.03)	1.02 (1.02, 1.02)	1.01 (1.01, 1.02)	1.09 (1.09, 1.10)	1.40 (1.37, 1.42)	1.13 (1.12, 1.13)
B2: Further adjusting for temperature	1,232,312	1.01 (1.01, 1.02)	1.01 (1.00, 1.01)	0.99 (0.99, 1.00)	1.08 (1.08, 1.09)	1.23 (1.20, 1.26)	1.12 (1.10, 1.12)
B3: Further adjusting for TT3 & TT4	83,7474	1.05 (1.05, 1.05)	1.02 (1.02, 1.02)	1.05 (1.05, 1.06)	1.09 (1.09, 1.10)	1.34 (1.32, 1.37)	1.10 (1.09,1.10)

a Each model was basically adjusted for age, sex, fasting blood glucose, body mass index, triglyceride, low density lipoprotein, high density lipoprotein, Urine iodine, O3, Gross Domestic Product (GDP), education index and average smoking rate. P-values for trend in all models were all less than 0.05.

Only 1.0% of participants had available thyroid autoantibody results (**Supplementary Table 3**). Analyses restricted to participants with anti-TG (+) and anti-TPO (+) and those with anti-TG (−) and anti-TPO (−) resulted in the slightly smaller ORs for all pollutants than the ORs from the main analyses including all participants. Most of the ORs turned to be not significant, except for CO and O_3_. ORs were both significantly larger for CO (2.27, 95% CI: 2.10–2.46) and O_3_ (1.38, 95% CI: 1.36–1.42) in anti-TG (−) and anti-TPO (−) groups (**Supplementary Table 2**).

### Threshold Analyses and Nonlinear Tests for Air Pollutants

**Figure 3:**
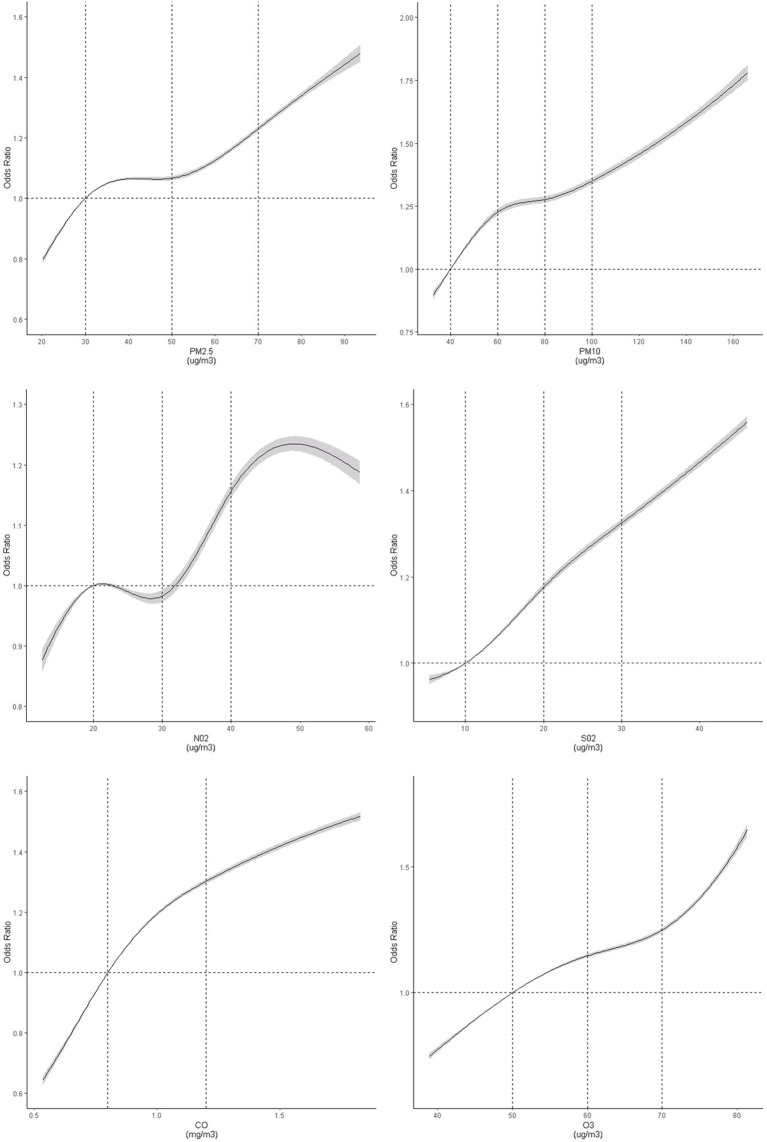
Dose–response association between air pollutants and risk of thyroid nodules. Restricted cubic spline was fitted for each air pollutant concentrations and thyroid nodules, with selected knots and reference for each air pollutants (PM_2.5_: 30 [Ref], 50, 70 ug/m^3^; PM_10_: 40 [Ref], 60, 80, 100 ug/m^3^; NO_2_: [20], 30, 40 ug/m^3^; SO_2_: 10 [Ref], 20, 30 ug/m^3^; CO: 0.8[Ref], 1.2 mg/m^3^; O_3_: 40[Ref], 50, 60, 70 ug/m^3^). The model was adjusted for age, sex, fasting blood glucose, body mass index, triglyceride, low density lipoprotein, high density lipoprotein, urine iodine, O_3_, Gross Domestic Product, education index, and average smoking rate. The graph indicated a non-linear association between air pollutant concentrations and thyroid nodules (test for spline model: *P <*0.0001). *P*-values for nonlinear tests were 0.0029, <0.0001, 0.0144, 0.9585, 0.0118 and 0.21473 for six pollutants, respectively.

In the threshold analyses, ORs for TN increased significantly when people were exposed to NO_2_ concentration of below 25 μg/m^3^, while ORs for TN kept rising significantly when only including participants exposed to the NO_2_ concentration of below 0.70 mg/m^3^. The ORs for TN were not significant when participants within either SO_2_ less than 10 μg/m^3^ or O_3_ less than 50 μg/m^3^ were restricted. These findings were complemented by the results of the spline models, which showed that the association did not deviate significantly from a linear association (**Figure 3**). Any rise in each of the air pollutant exposure was associated with an elevated thyroid nodules risk.

## Discussion

As mentioned before, there has been no prior study that has extensively discussed the potential risk that outdoor air pollutants have on the increasing prevalence of TNs. This study found that each of the six major air pollutants (PM_2.5_, PM_10_, NO_2_, SO_2_, CO, and O_3_) were statistically associated with an increased risk of TNs among about 4.9 million Chinese adults. These associations were significantly stronger in the participants, who were men, younger, and also having lower urine iodine level. Those associations were close to linear in all and subgroup analyses, indicating that air pollutants exposure was associated with an elevated TNs risk without minimum effective concentrations.

The association between major air pollutants and TNs was near linear without minimum effect concentrations. The linear results for PM_2.5_ and PM_10_ were indeed unsurprising because the minimum levels for both the two pollutants in this study exceeded the level (minimum 10 μg/m^3^ vs. 20 μg/m^3^) suggested by the WHO air quality guideline (18). However, it was unexpected for NO_2_, SO_2_, CO, O_3_, and ORs to remain statistically significantly heightened when only participants with concentrations far below corresponding the WHO air quality guideline (<25 μg/m^3^ for NO_2_, 10 μg/m^3^ for SO_2_, 700 μg/m^3^ for CO, and 60 μg/m^3^ for O_3_) were included. The current WHO annual mean limit value for NO_2_, SO_2_, CO, and O_3_ were 40 μg/m^3^, 20 μg/m^3^, 4,000 μg/m^3^, 100 μg/m^3^, respectively. These findings suggest that significant adverse health effects occur at concentrations well below accepted limits, supporting the idea that significant health benefits can be achieved by moving towards that guideline, while also adding new evidence concerning the important human organ.

The significant associations between air pollutants exposure and TNs remained stable in this study, even after thoroughly adjusting well-known confounders adhering to previous studies (9, 21). Those findings were complementarily consistent with existing sets of evidence focused on the association between air pollutants and thyroid dysfunction (6–9). The potential mechanisms explaining the effect of outdoor air pollutants on TNs risk are still unclear. Several signaling pathways may be involved. First, the outdoor air pollutants exposure may cause oxidative stress in the body, which has been described as an endogenous factor contributing to the thyroid hyperplasia (22). Second, exposure to particulate matters shapes DNA methylation through the lifespan (23), which may also be a potential mechanism for TNs pathogenesis. In addition, other pathways such as systemic inflammation and insulin resistance might also be among potential mechanisms (24, 25).

Furthermore, it was found that the association of air pollutants was ameliorated with TSH adjustment. TSH has been proposed to promote growth in size of thyroid cells (26). In this study, the TSH levels were relatively lower in the TNs group, which might be due to its ability to maintain TSH levels at a lower normal range by autonomous hyperfunctioning (27). Thyroid antibodies might also contribute to the growth and progression of TN (28). In this study, most of the ORs turned to be nonsignificant in anti-TG(+) and anti-TPO(+) group except for CO and O_3_. One possible reason was that the effects of anti-TG and anti-TPO on TNs progression were too high that the effect of air pollutants was masked. Another possible reason was that the anti-TG(+) and anti-TPO(+) patients have more sophisticated immune systems compared to the anti-TG(−) and anti-TPO(−) groups, which may have a stronger inflammatory response when their immune systems are triggered, and the moderate inflammation may play some role in defending air pollutant effect. Some studies also reported that high anti-TPO titers appear to protect against differentiated thyroid cancer in patients with Hashimoto’s thyroiditis (29).

Females have been reported to be more vulnerable to air pollution (PM_10_, SO_2_, NO_2_, and O_3_), and to have a higher air pollution-related mortality than males in a few studies (30). However, this study with a large sample size demonstrated that ORs for each pollutant with TNs were consistently lower in participants who were women and/or elderly, which contradicts the sex-specific effect of air pollutant on other outcomes including thyroid function indexes and mortality. Clear reasons for the sex-specific effects of air pollution are not well known. One explanation could be due to the fact that women and the elderly were already more likely to suffer from TNs (5), while the group with higher risk profiles is always apt to detect a relatively lower OR resulting from some loss of statistical power (20). Another reason is that the estrogen and progesterone might also contribute to the sex disparity (31), which needs further study to confirm. Another interesting discovery in this study was that lower urine iodine level has higher ORs for each pollutant. The relatively low risk profile in the group with appropriate urine iodine level should also be one reason. Besides, cigarette smoking could inhibit iodine transport, iodine organification, and increase iodide efflux, which may promote the occurrence of thyroid nodules (32). It is reasonable to postulate that the effect of air pollutant on iodine metabolism is ameliorated in the excess iodine region.

Air pollutants especially particulate matter in outdoor air pollution has become one of the biggest threats to the health of the Chinese people. Although appreciable reductions of the annual average concentrations for most of pollutants were observed from 2013 (33), the current concentrations for fine particulate, NO_2_ and SO_2_ were still higher than the WHO recommendations. The newly-found associations together with previous researches (6–9), have at least two implications. First, the new evidence was added for the adverse health effect of air pollution in the perspective of the important endocrine organ, with connecting major air pollutants with the highly prevalent disease. Second, it is safely assumed that plenty of TNs patients would unsurprisingly appear in next decades without expeditious and effective measures to control air pollutants. The findings provide evidence to implement effective air-quality improvement strategies and management plans, addressing an arduous task in managing the challenges of environmental pollution in China and other countries facing similar air pollution issues.

Nonetheless, the study has several limitations. First, assigning each participant the average concentration of air pollutants over the city in which he or she visited the health screening center was a rough estimate, but the corresponding variation is expected to be minimal. Second, whether TNs were benign or cancerous were not diagnosed by the general practice in health screening centers, which has hindered further exploration of the relationship between air pollutants and the different types of TNs (21). Lastly, causal inference was limited by the observational study design, although the reverse effect of TNs causing air pollutants changes is scarcely possible.

## Conclusions

In conclusion, with an unprecedented large sample size and measures of multiple air pollutants across a wide geographic area, the findings of this study show that exposure to six air pollutants is significantly associated with an increased risk of thyroids nodules, even at concentration ranges well below the annual mean limit value suggested by the WHO. Further longitudinal studies are needed to confirm these findings, and to clarify potential mechanisms that might be responsible for the association between air pollutants exposure and TNs.

## Data Availability Statement

The raw data supporting the conclusions of this article will be made available by the authors, without undue reservation.

## Ethics Statement

The studies involving human participants were reviewed and approved by the Ethical Committee of the Health Science Center of Peking University. The patients/participants provided their written informed consent to participate in this study.

## Author Contributions

Conception: SW, YFS, and YN. Design: SW, YFS, and YN. Administrative support: YN and LL. Provision of study material or patients: CJ. Collection and assembly of data: YZ, WQ, SW, and CJ. Data analysis and interpretation: YZ, KW, SW, YFS, YQS, PJ, YN, and LL. Manuscript writing: YZ, KW, SW, and YFS. All authors contributed to the article and approved the submitted version.

## Funding

This work was funded by the National Natural Science Foundation of China (81922016, 91846303, and 81502884) and the National Key R&D program (2020YFC2003400). The funders had no role in the study design; collection, analysis, and interpretation of data; writing of the report; or decision to submit the article for publication.

## Conflict of Interest

The authors declare that the research was conducted in the absence of any commercial or financial relationships that could be construed as a potential conflict of interest.

## Publisher’s Note

All claims expressed in this article are solely those of the authors and do not necessarily represent those of their affiliated organizations, or those of the publisher, the editors and the reviewers. Any product that may be evaluated in this article, or claim that may be made by its manufacturer, is not guaranteed or endorsed by the publisher.
