# Supplementary material for: Six Air Pollutants Associated With Increased Risk of Thyroid Nodules: A Study of 4.9 Million Chinese Adults
Source: Front Endocrinol (Lausanne). 2021 Dec 13;12:753607. doi: 10.3389/fendo.2021.753607 (PMC8710776; doi:10.3389/fendo.2021.753607)
Supplement: Supplementary file 1 [file Table_1.docx]

## Supplementary Table 1. Pearson correlation coefficients between PM_2.5_, PM_10_, NO_2_, SO_2_, CO, and O_3_ average concentrations in 2017 in 157 cities included in the study

|  | **PM_2.5_** | **PM_10_** | **NO_2_** | **SO_2_** | **CO** | **O_3_** |
| --- | --- | --- | --- | --- | --- | --- |
| **PM_2.5_** | 1.00 | 0.90** | 0.64** | 0.47** | 0.61** | 0.13 |
| **PM_10_** | - | 1.00 | 0.55** | 0.45** | 0.61** | 0.16 |
| **NO_2_** | - | - | 1.00 | 0.41** | 0.48** | -0.05 |
| **SO_2_** | - | - | - | 1.00 | 0.63** | 0.13 |
| **CO** | - | - | - | - | 1.00 | -0.07 |
| **O_3_** | - | - | - | - | - | 1.00 |

Note: ** mean means *P*< 0.01. All other *P* values are larger than 0.05.

## Supplementary Table 2. Sensitivity analyses results^a^

|  | | **A: Adjust the potential effect**  **of city heterogeneity (N=4920536)** |  | **Sample size ^b^** | **B1: Adjusting for thyroid**  **stimulating hormone** | **B2: Further adjusting for temperature** | **Sample size** | **B3: Further adjusting for TT3 & TT4** |
| --- | --- | --- | --- | --- | --- | --- | --- | --- |
| PM2.5, ug/m^3^ |  |  |  |  |  |  |  |  |
|  | 10~29 | 1.00(ref) |  | 158,890 | 1.00(ref) | 1.00 (ref) | 165,617 | 1.00 (ref) |
|  | 30~49 | 1.08 (0.90, 1.30) |  | 437,431 | 1.15 (1.13,1.17) | 1.11 (1.09, 1.13) | 234,,135 | 1.20 (1.18,1.22) |
|  | 50~69 | 1.16 (0.96, 1.41) |  | 506,863 | 1.16 (1.14, 1.17) | 1.07 (1.05, 1.09) | 328,118 | 1.26 (1.24,1.28) |
|  | 70~ | 1.34 (1.07, 1.69) |  | 129,128 | 1.28 (1.25, 1.30) | 1.19 (1.16, 1.21) | 109,604 | 1.36 (1.33,1.39) |
| PM10, ug/m^3^ |  |  |  |  |  |  |  |  |
|  | 20~39 | 1.00(ref) |  | 19,611 | 1.00(ref) | 1.00 (ref) | 20,544 | 1.00 (ref) |
|  | 40~59 | 0.97 (0.66, 1.43) |  | 203,229 | 1.33 (1.28, 1.38) | 1.32 (1.27, 1.37) | 168,921 | 1.32 (1.28,1.37) |
|  | 60~79 | 1.02 (0.70, 1.48) |  | 362,294 | 1.36 (1.32, 1.41) | 1.26 (1.21, 1.31) | 207,652 | 1.42 (1.38,1.47) |
|  | 80~99 | 1.10 (0.75, 1.60) |  | 386,023 | 1.43 (1.39, 1.48) | 1.30 (1.25, 1.34) | 239,804 | 1.62 (1.57,1.68) |
|  | 100~ | 1.21 (0.83, 1.79) |  | 261,155 | 1.49 (1.44, 1.54) | 1.29 (1.24, 1.33) | 200,553 | 1.52 (1.47,1.57) |
| NO2, ug/m^3^ |  |  |  |  |  |  |  |  |
|  | <20 | 1.00(ref) |  | 33,053 | 1.00(ref) | 1.00 (ref) | 31,988 | 1.00 (ref) |
|  | 20~29 | 1.30 (1.02, 1.65) |  | 168,478 | 1.44 (1.40, 1.48) | 1.21 (1.17, 1.24) | 82,778 | 1.45 (1.41,1.49) |
|  | 30~39 | 1.32 (1.03, 1.68) |  | 231,372 | 1.46 (1.42, 1.50) | 1.30 (1.26, 1.34) | 184,866 | 1.55 (1.51,1.60) |
|  | 40~ | 1.55 (1.20, 2.00) |  | 799,409 | 1.40 (1.37, 1.44) | 1.20 (1.17, 1.24) | 537,842 | 1.54 (1.50,1.58) |
| SO2, ug/m^3^ |  |  |  |  |  |  |  |  |
|  | <10 | 1.00(ref) |  | 164,359 | 1.00(ref) | 1.00 (ref) | 162,932 | 1.00 (ref) |
|  | 10~19 | 1.04 (0.87, 1.24) |  | 673,505 | 1.10 (1.08, 1.11) | 1.10 (1.09, 1.12) | 429,641 | 1.17 (1.15,1.18) |
|  | 20~29 | 1.17 (0.97, 1.40) |  | 241,549 | 1.11 (1.10, 1.13) | 1.08 (1.06, 1.10) | 133,878 | 0.99 (0.98,1.01) |
|  | 30~ | 1.33 (1.11, 1.60) |  | 152,899 | 1.56 (1.53, 1.59) | 1.52 (1.49, 1.55) | 111,023 | 1.63 (1.60,1.66) |
| CO, mg/m^3^ |  |  |  |  |  |  |  |  |
|  | 0.40~0.79 | 1.00(ref) |  | 182,291 | 1.00(ref) | 1.00 (ref) | 168,319 | 1.00 (ref) |
|  | 0.80~1.19 | 1.16 (0.99, 1.36) |  | 778,562 | 1.22 (1.21, 1.24) | 1.23 (1.21, 1.25) | 464,004 | 1.24 (1.22,1.25) |
|  | 1.20~ | 1.33(1.12, 1.59) |  | 271,456 | 1.37 (1.35, 1.39) | 1.31 (1.29, 1.33) | 205,151 | 1.31 (1.29,1.33) |
| O3, ug/m^3^ |  |  |  |  |  |  |  |  |
|  | <50 | 1.00(ref) |  | 195,525 | 1.00(ref) | 1.00 (ref) | 111,002 | 1.00 (ref) |
|  | 50~59 | 1.17 (1.00, 1.36) |  | 627,115 | 1.18 (1.16, 1.19) | 1.18 (1.17, 1.20) | 434,967 | 1.31 (1.29,1.33) |
|  | 60~69 | 1.37 (1.17, 1.62) |  | 186,178 | 1.34 (1.32, 1.36) | 1.34 (1.32, 1.36) | 156,658 | 1.38 (1.36,1.41) |
|  | 70~ | 1.45 (1.18, 1.79) |  | 223,494 | 1.38 (1.36, 1.40) | 1.34 (1.32, 1.36) | 134,847 | 1.39 (1.36,1.42) |

Note: ^a^Each model was basically adjusted for age, sex, fasting blood glucose, body mass index, triglyceride, low density lipoprotein, high density lipoprotein, Urine iodine, O3, Gross Domestic Product (GDP), education index and average smoking rate. And *P* values for trend in all models were all less than 0.05.

^b^Only observations with complete information for model variables were included in the analyses.

## Supplementary Table 3. Results of Logistic regression for the association between exposure to air pollution and thyroid nodules, stratified by status of thyroglobulin antibodies (anti-TG) and anti-thyroid peroxidase antibody (anti-TPO)

|  | **anti-TG (+) & anti-TPO (+)** | **anti-TG (-) & anti-TPO (-)** | **Wald χ^2^** | ***P* value** |
| --- | --- | --- | --- | --- |
| N | 6051 | 44929 |  |  |
| PM_2.5_, per 10 ug/m^3^ | 1.04 (0.99, 1.09) | 1.04 (1.02, 1.06) | 0.0315 | 0.86 |
| PM_10_, per 10 ug/m^3^ | 1.02 (0.99, 1.04) | 1.00 (0.99, 1.01) | 0.8109 | 0.37 |
| NO_2_, per 10 ug/m^3^ | 1.00 (0.95, 1.10) | 1.05 (1.02, 1.08) | 1.1696 | 0.28 |
| SO_2_, per 10 ug/m^3^ | 1.04 (0.98, 1.10) | 0.89 (0.87, 0.90) | 2.4245 | 0.12 |
| CO, per mg/m^3^ | 1.15 (0.92, 1.40) | 2.27 (2.10, 2.46) | 12.1984 | 0.0005 |
| O_3_, per 10 ug/m^3^ | 1.14 (1.07, 1.21) | 1.38 (1.36, 1.42) | 54.4820 | <0.0001 |

Note: Model was basically adjusted for age, sex, fasting blood glucose, body mass index, triglyceride, low density lipoprotein, high density lipoprotein, Urine iodine, O3, Gross Domestic Product (GDP), education index and average smoking rate.
